# Lymphatic Obstruction Related to Small Bowel Obstruction With Chylous Ascites in Prior Roux En Y Gastric Bypass Patient Case Report

**DOI:** 10.1155/cris/6093542

**Published:** 2025-08-29

**Authors:** Caroline Couch, Jonathan Chica

**Affiliations:** Department of Trauma, Allegheny Health Network, Pittsburgh, Pennsylvania, USA

## Abstract

Chylous ascites from small bowel obstructions is a very rare finding with only a handful of case reports previously published. This case report of a patient with chylous ascites related to an obstruction from Petersen's hernia supports the trend from existing reports. Prior studies have linked chylous ascites to closed-loop obstructions, such as small bowel volvulus or internal hernia, even when the bowel is viable and does not require resection.

## 1. Introduction

Small bowel obstructions are one of the most common problems encountered in general surgery, and it is well known that threatened bowel can present with serous or bloody ascites. Chylous ascites is a much more uncommon scenario. It is usually associated with an injury to a lymphatic channel or invasion of lymphatic ducts from cancer [1]. However, chylous ascites has been reported in patients with small bowel obstructions. The incidence of this occurrence has been limited to case reports [2–5]. Upon a review of the literature, there was one case report of a patient who developed chylous ascites secondary to a volvulus from a Petersen's hernia after a Roux-en-Y gastric bypass (RYGB) [2]. Given the rarity of this finding, we report a similar case of chylous ascites after a Petersen's internal hernia to add to the limited information available on this occurrence and support the reported theory that chylous ascites can be an indicator of bowel viability [4].

## 2. Case Presentation

**Figure 1:**
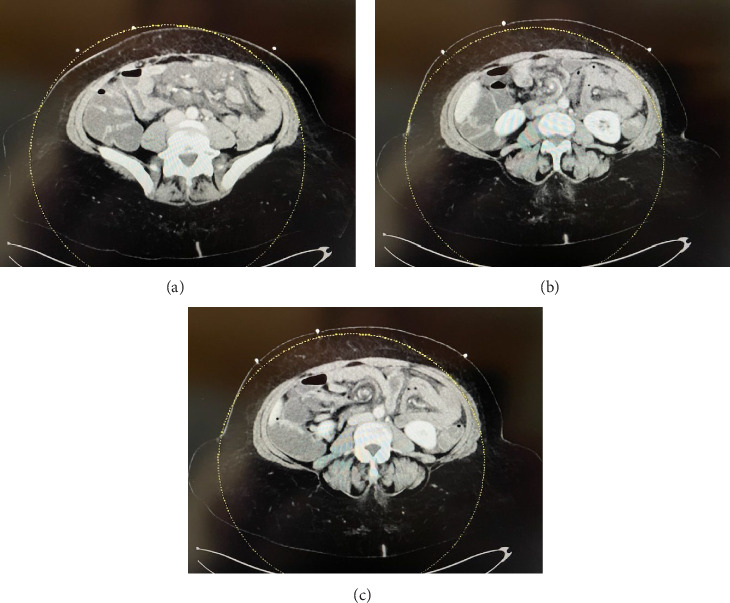
Preoperative CT scan. (a) Pelvis, (b) lower abdomen, and (c) mid abdomen.

A 56-year-old female with a remote history of a RYGB presented to the emergency department with a 4-day history of abdominal pain, nausea, and vomiting, which worsened over the last 48 h. Her labs showed an elevated lactate, and on physical exam, she had signs of peritonitis. Her CT imaging workup showed hypoenhancement of the jejunum with concern for impingement of the distal SMA and a small volume of free fluid in the pelvis that was new from prior CT scans. She still had a swirling of the mesentery that had been visible on her prior CT scan (Figure 1). No prior record of her RYGB was able to be obtained due to the many years since the operation and the fact that it was performed at an outside hospital. A nasogastric tube was placed, and she was taken emergently to the operating room for exploratory laparotomy.

Upon entering the abdomen, milky white ascites was encountered, and almost the entire small intestine showed venous congestion with a very edematous mesentery with lymphatic channels clearly visible, containing similarly colored white fluid. The bowel was traced and found to be contained in an internal hernia through a Petersen's mesenteric defect that was approximately 10 cm in size. There was no suture visible to suggest a prior closure of the defect or closure with absorbable sutures. The hernia was reduced, removing at least 60 cm of the common channel small bowel from the hernia defect. The Petersen's mesenteric defect was then closed with silk suture. There was no mass or injury to the bowel or retroperitoneum as an alternative source of the chylous ascites. The appearance of the bowel improved during the operation, and no bowel resection was required.

Postoperatively the patient recovered uneventfully. She regained bowel function, tolerated a diet, and was discharged on postoperative day six. She was seen at 1 and 2 weeks postoperatively and was healing well.

## 3. Discussion

Chylous ascites is a rare occurrence that is most associated with cancer that invades the abdominal lymphatic system. Traumatic and iatrogenic injuries to the lymphatic system are also sources of chylous ascites. However, there are documented cases secondary to bowel obstructions [2–5]. Only one other study reports chylous ascites related to a Petersen's hernia [2]. The theory established by prior papers is that chylous ascites may form when pressure from the bowel obstruction, such as in cases of volvulus or internal hernia, obstructs normal lymphatic flow. The pressure prevents the normal flow of lymph from the bowel into the lymphatic ducts, so it is exuded from the bowel and lymphatic ducts into the abdomen. During surgery, this phenomenon was observed in our patient as evidenced by milky fluid in the ascites and in the dilated lymphatics on the bowel and mesentery. It has also been theorized that the presence of chylous ascites predicts salvageable bowel as the pressure to obstruct lymphatics is lower than that to obstruct vascular flow and cause bowel ischemia [4]. This theory of bowel viability is also supported by this case report, as the patient did not have to have any bowel resected despite a very large portion of her bowel herniated through the defect. None of the cited case reports [2–5] required bowel resection related to the bowel obstructions. While this is a single case report, it highlights a very rare occurrence only seen in a few other case reports and supports the theories established in that literature that chylous ascites can form related to a Petersen's hernia and that bowel involved in the small bowel obstruction is normally viable. Of note, this patient also had no signs of prior closure of the Petersen's mesenteric defect with permanent suture. Prior studies [6] suggest that closure of that defect could have reduced the risk of internal hernia from 15.5% to 6.5% at 5 years postsurgery.

## 4. Conclusion

This case report illustrates and confirms the existing case reports that patients with closed-loop bowel obstructions and particularly internal hernias can develop chylous ascites in rare cases. This chylous ascites seems to be a positive prognostic indicator as the blockage of the lymphatics occurs at a lower pressure than vascular compromise, thus the involved bowel in our case and prior cases has not required resection.

## Data Availability

Data sharing is not applicable to this article as no datasets were generated or analyzed during the current study.
